# Keep Your Feet Warm

**Published:** 1875-02

**Authors:** 


### KEEP YOUR FEET WARM.

We have probably said this a million times if we include all our utterances by tongue and pen and types. But it has evidently amounted to nothing. People muffle up the head and throat and chest with numerous thicknesses of cloth and fur, and leave only a thin film of animal fibre between their feet and the ice and snow. They treat their feet as if they were not a part of the individual at all; as if they were merely to be shod like the foot of a horse, or the tip of a cane or umbrella, to keep them from wearing out. They clearly forget the intimate connection between the feet and the heart, lungs, liver, and all the vital parts. They lose sight of the fact that the life-current—the blood—can be impeded as surely by cold feet as by strong ligatures, and that to so impede the circulation is a very dangerous thing to do. It seems hard to make people understand that they by no means save the lungs from disease when they cover them up with many layers of protecting substance. Not once in a million times is a cold taken "on the lungs" by a lack of clothing over the region of the lungs. Lung diseases spring from many sources, but rarely, perhaps never, from lung-exposure. If the strong, bony frame-work, the marvelous muscular system and the covering of tissues, were all removed from the lungs, there might be some sense in muffling and binding the chest in the fashionable winter style; but as this rarely occurs, it is clear that the chest needs no covering save such as is demanded by the rest of the trunk, and to retain the natural heat. The abdomen and the loins certainly demand greater protection than the chest, although they never get it. All sorts of pads and "protectors" are exposed for sale to keep the air away from "the lungs," as it is called; when the truth is that the lungs suffer more in one hour from the lowering of the temperature of the blood by cold feet, than they would by the exposure of the surface of the chest to the most chilling blasts for a whole day.

The feet must be kept dry and warm, and not only the feet but the legs. Congestion of the lungs, acute pneumonia, lung fever, bronchitis, and all manner of throat and chest diseases, will follow chilled extremities far oftener than they will the exposure of the chest to the winter blasts. So in putting on furs and shawls and mufflers, remember that the feet are to be muffled first, and that to forget to muffle the throat will do no very great deal of harm. The feet must be kept warm, or there will be trouble in one direction or another.

---

MANY a man and many a woman, who has rather feeble digestive powers, passes a somewhat disturbed or sleepless night, and gets up in the morning feeling "poorly." Indigestion is the trouble, though such is not generally supposed to be the fact. It can be prevented by light eating at night. Let the full meal come in the middle of the day. Two hours after it has been taken, walk three or four miles. Eat a light, easily-digested supper, and pass the succeeding hours till bed-time in an agreeable but not exciting manner. Avoid causes of worry, sleep in a fresh bed and well ventilated apartments, and retire in good time.

---

The idea of making flour by crushing the grain with innumerable small triphamners has been carried out in England so successfully that a pounding mill costing $1,000 will produce as much flour in the same time as a grinding mill worth $5,000.

